# Spray drying of pomegranate juice using maltodextrin/cyclodextrin blends as the wall material

**DOI:** 10.1002/fsn3.467

**Published:** 2017-03-01

**Authors:** Michael A. Watson, Jeanne M. Lea, Karen L. Bett‐Garber

**Affiliations:** ^1^ U.S. Department of AgricultureSouthern Regional Research CenterNew OrleansLouisiana

**Keywords:** Pomegranate juice, spray drying, astringency, γ‐cyclodextrin, maltodextrin

## Abstract

Microencapsulation protects sensitive nutrients, masks flavors, or enhances delivery. Ratios of maltodextrin and γ‐cyclodextrin (20:0, 19:1, and 17:3% w/w) were dissolved in water and mixed with pomegranate juice for spray drying with inlet temperatures of 120, 140, and 160°C. The effects on physical properties (water activity, % water content, color, pH, soluble solids (Brix), and methyl cellulose precipitable tannin assay (MCPTA) were examined. Based on the principle component analysis, formulation influenced color parameters and pH accounted for 46.8% of the variation in the data. Temperature influenced Chroma and water‐holding capacity with 31.8% of the variation. The pH of the reconstituted spray‐dried powder significantly influenced color. Blending of γ‐cyclodextrins to maltodextrins slightly increased the water‐holding capacity, increased pH, slightly affected color, and preserved the color over time, slightly better. Increased inlet temperature affected color, decreased water‐holding capacity, and decreased astringency index. Small additions of γ‐cyclodextrin affect spray‐dried powders.

## 1 Introduction

Encapsulation is a process in which flavors, nutrients, oils, proteins (i.e., enzymes, hormones), or probiotic bacteria are enveloped into a starch, protein, or lipid carrier matrix for preservation, masking, or delivery of the encapsulated agent. It can mask smell or taste, and it can mask pigments or the color of substances (Del Valle, 2004). The carrier matrix needs to be food grade (Food and Drug Administration, or European Food Safety Authority approved), provide maximal protection of the core material under harsh conditions, preserve the active molecule during processing or storage, decrease transfer rate of core material to the environment, mask core material taste, or dilute the core material when only small amounts are required (Alexe & Dima, 2014 and Gharsallaoui, Roudaut, Chambin, Voilley, & Saurel, 2007). Encapsulation can be accomplished by spray drying, extrusion, freeze drying, or fluid bed coating. Spray drying is one of the oldest and widely used methods. Its advantage is that it is easily applied on an industrial scale. It requires complex equipment, and is limited to carrier matrix material that is soluble or dispersible in water (Alexe & Dima, 2014).

Spray drying has three fundamental steps, atomization of the liquid feed, mixing fine droplets with heated air stream to dry, and separation of dried particles from air stream for collection. Modifying the process parameters can alter properties, such as particle size, particle shape, flowability, bulk density, solubility, moisture content, thermal stability, or suitability for food applications (Mahdavi, Jafari, Ghorbani, & Assadpoor, 2014). Encapsulation should result in powder with low surface pigment and maximum retention of the colorant in the core. The optimal condition for natural anthocyanins is a compromise between high air temperature, high solids concentration of the solution, and little expansion or cracking during drying. Spray drying accounts for 80–90% of encapsulated anthocyanins (Mahdavi et al., 2014).

Wall material is selected based on its ability to protect the core material from environments that cause deterioration or protect and increase the stability of the material. The wall material is selected for properties such as solubility, molecular weight, diffusibility, and emulsion properties (Kandansamy & Somasundaram, 2012), and is a low‐molecular weight starch, gum, or protein. Hydroscopicity is also a key property for selecting a wall material (Raja, Sankarikutty, Sreekumar, Jayalekshmy, & Narayanan, 1989). Raja et al. (1989) declared that maltodextrins with a dextrose equivalent (DE) of 10–20 worked best for wall materials in spray‐dried powders due to less turbidity at high concentrations. The low haze formation of low DE makes for more uniform coating and greater preservation of coated material. Peng, Li, Guan, and Zhao (2013) observed that maltodextrin wall material was superior to β‐cyclodextrin for protecting the antioxidant components. The material also needs to be inexpensive, food grade, readily available, and legally allowed (Mahdavi et al., 2014). Partanen, Ahro, Hakala, Kallio, and Forssell (2002) observed that maltodextrin was more heat stable than β–cyclodextrin under dry conditions.

Cyclodextrins are cyclic oligosaccharides produced by the degradation of starch resulting from intramolecular transglycosylation reactions caused by cyclodextrin glucanotransferase enzyme. There are several types—α‐cyclodextrin which have six glucose molecules in the ring, β‐cyclodextrin which have seven glucose molecules in the ring, and γ‐cyclodextrin, which have eight or more glucose units (Del Valle, 2004). The height of the cyclodextrin cavity is the same for all three types but the diameter varies with the number of glucose units. Therefore, the volume depends on the number of glucose units. Selection of the type of cyclodextrin depends on the size of molecule to be included. Small molecules are included in α‐cyclodextrin, whereas larger molecules are included in γ‐cyclodextrin. In addition to size, inclusion depends on thermodynamic interactions. A favorable net energetic driving force is needed to pull the guest molecule into the cyclodextrin cavity (Del Valle, 2004). Along with the active material, the equilibrium kinetics, other formulation ingredients, and processes utilized, some water is needed to drive the thermodynamics to form the inclusion complexes. A variety of noncovalent forces are responsible for a stable inclusion, such as van der Waals forces. More than one cyclodextrin can form a complex around the inclusion molecule. Inclusion occurs more readily when the guest molecule is in soluble form. Heating the solution can destabilize the complex, and most complexes start to decompose at around 50–60°C (Del Valle, 2004). As mentioned above, some water is needed to drive thermodynamic interactions, but ample water increased the solubility of the cyclodextrin and the guest molecule. Too much water decreases the odds of the cyclodextrin and the guest molecule coming into contact, and slows the rate of complexation (Del Valle, 2004). γ‐Cyclodextrins have greater internal cavities, are more water soluble, and allows for the inclusion to be more bioavailable (Li et al., 2007). γ ‐Cyclodextrins can accommodate much larger molecules, such as, macrocycles and steroids (Del Valle, 2004). γ ‐Cyclodextrins can be absorbed by the human intestines which make it ideal for food applications (Li et al., 2007). It can achieve a nearly complete inclusion of a molecule, which can protect the inclusion molecule from autoxidation during storage (Koeda et al., 2014; O'Donnell, 2001).

Mixtures of encapsulating compounds: Encapsulating the complex of compounds in food, for example, a juice may take multiple complexation materials. Many complexing mixtures include maltodextrins, cyclodextrins, gums, such as gum arabic, and protein isolates, such as whey and soy (Donovan, Cadwallader, & Lee, 2016). γ‐Cyclodextrin has been shown to increase the capacity of carriers (Duchene, Ponchel, & Wouessidjewe, 1999). Koeda et al. (2014) observed that a mixture of α‐cyclodextrin and maltodextrin exhibited greater stability of retinyl palmitate, than with β‐ and γ‐cyclodextrin. Inulin combined with whey protein concentrate resulted in fewer surface cracks, along with a smoother surface (Donovan et al., 2016).

Inlet temperature: Typically, when feed temperature is increased, then the viscosity and droplet size is decreased. Also, drying rate is increased. This results in a finer powder. Although, Chong et al. (2014) observed that spray drying with maltodextrin at higher drying temperature and faster drying rate resulted in larger droplet sizes. They theorized that the fast drying rate restricted the shrinkage of the droplets during drying. If encapsulating a volatile compound, higher temperatures lead to loss of volatiles (Gharsallaoui et al., 2007; Chong et al., 2014). Spray drying is the method of choice for heat‐sensitive foods. Therefore, there is a balance between inlet temperature and preservation of the encapsulated compound (Rattes & Oliveira, 2007) of which compounds in pomegranate juice can be sensitive. At higher drying temperatures, the spray‐dried product results in lower moisture contents (Rattes & Oliveira, 2007).

In this study, we will evaluate the effectiveness of γ‐cyclodextrins and maltodextrins as wall material for spray‐dried pomegranate juice. The effect of wall material and drying temperature on physical characteristics of the powders and reconstituted solution will be examined.

## 2 Materials and methods

### 2.1 Fresh pomegranate juice

Lakewood Organic Pure Pomegranate juice‐Fresh pressed (Lakewood, Miami, FL) was obtained from local Whole Foods Market. The juice obtained was organic and not reconstituted.

Pomegranate juice was mixed with a maltodextrin/γ‐cyclodextrin combination. The concentration of maltodextrin with a dextrose equivalent (DE) of 12 (Glucidex 12, Roquette Chemical, Lestrem, France) was 17%, 19%, and 20% respectively. The concentration of γ‐cyclodextrin (Wacker Chemical AG, Munchen, Germany) was 0%, 1%, and 3%, respectively.

Approximately, 400 g deionized water was added to a beaker and heated to 50°C. For each respective solution, maltodextrin was added slowly and allowed to stir until complete dissolution. Next, γ‐cyclodextrin was added to each respective solution and allowed to dissolve completely. Approximately, 200 g of pomegranate juice was added to each respective concentration mixture and allowed to stir for 1 hr. After 1 hr stirring with a magnetic stir bar, each respective mixture was homogenized for 3 min using a Tissuemizer (Tekmar, Cincinnati, OH). Each respective mixture was then ready for further processing.

The solutions resulting from this homogenization were then fed into a Yamato Model GB 22 Spray Dryer (SD) with the following conditions: The SD operating conditions were—inlet air temperature was 120°C, 140°C, and 160°C; maltodextrin and γ‐cyclodextrins concentration was 0, 1, and 3%, respectively; feed rate of 13 ml/min; outlet air temperature ranged from 70°C to 80°C.

### 2.2 Spray drying conditions

Maltodextrin (DE 12) and γ‐cyclodextrin were selected for the wall material because maltodextrin with low dextrose equivalent and γ‐cyclodextrin are better at protecting antioxidents than β‐cyclodextrin (Koeda et al., 2014 and O'Donnell, 2001). Each feed mixture of maltodextrins and/or maltodextrin‐cyclodextrin concentrations were spray dried using a Yamato Pulvis GB 22 Spray dryer (Orangeburg, NY). The inlet temperatures were 120°C, 140°C, and 160°C. The drying air flow rate, compressor air pressure (0.45 m^3^/min), and feed rate were constant. After spray drying, the samples were stored in Brown UV 500 ml bottles and placed in −20 degree freezer until analyzed.

### 2.3 Moisture content

The moisture content of spray‐dried pomegranate samples were determined by drying at the temperature of 105°C in the oven until a constant weight was obtained (Pitalua, Jimenez, Vernon‐Carter, & Beristain, 2010).

### 2.4 Water activity

A water activity meter (AquaLab PawKit series 3te, Decagon Devices, Pullman, Washington, USA) was used to measure water activity of the spray‐dried powders.

### 2.5 pH determination

The pH Unifit microprocessor Model UF100‐01 (San Diego, CA) value of Pomegranate powders was determined for pH by blending 5 g powder with 25 ml. deionized water at 20°C, using the pH meter calibrated with standard buffers pH 7 and 4.

### 2.6 Color

The color of Pomegranate juice powder sample was determined using a Konica‐Minolta Chroma meter CR‐410 (Konica‐Minolta Sensing, Tokyo, Japan) calibrated with a white standard tile. The results were expressed as hunter color values of L*, a*, and b*, where L* was used to denote lightness, a* redness and greenness, and b* yellowness and blueness. Chroma, hue angle (H°), and redness (a*/b*) were calculated according to Kha, Nguyen, and Roach (2010). Color was measured after spray drying and after 24 months of storage at −8.3 °C.

### 2.7 Soluble solids (Brix)

Approximately, 5‐g spray‐dried powder was reconstituted in 25 ml deionized water as described above. A small aliquot of this mixture was placed on the lens glass of a pocket refractometer Atago, PAL‐1 (Bellevue, WA) and Brix values were obtained.

### 2.8 Methyl cellulose precipitable tannin assay

The methyl cellulose precipitable tannin assay (MCPTA) method was used to assay the presence of condensed tannins as described in Mercurio, Dambergs, Herderich, and Smith (2007) and in Bett‐Garber et al. (2014). The MCPTA test was run on solutions of 5‐g spray‐dried powder reconstituted in 25 ml deionized water as described above.

### 2.9 Statistical methods

Statistical analysis was accomplished in SAS Enterprise Guide v 5.1. Analysis of variance was completed on the two‐way design using Proc Mixed. Means were compared with Tukey's HSD adjustment to LS Means. Pearson Correlation coefficients and significance were derived with Proc Corr. Principle component analysis was run with Proc Princomp.

## 3 Results and discussion

### 3.1 Color

The 3% γ‐cyclodextrin formulation across all three drying temperatures was less red (lower a*/b*) and less white (lower L* value) than the samples with less cyclodextrin content (Table 1). The 3% γ‐cyclodextrin also had greater hue angle (more yellow). The γ‐cyclodextrin appeared to mask the natural red color of the dried pomegranate juice. Comparing the air inlet temperature data across the three formulation (Table 1), temperature did not significantly affect L* value. Between the nine treatment combinations, the redness (a*/b*) was least in the 120°C‐dried and 3% γ‐cyclodextrin‐formulated powders (Table 2a), while the sample dried at 160°C with 0% γ‐cyclodextrin was the greater in a*/b*. The powder spray dried at 140°C, with the formulation of 1% γ‐cyclodextrin was the whitest color or largest L‐value of all nine treatment combinations. However, the powders dried at 140°C and 160°C for both 0% and 1% γ‐cyclodextrin were not significantly different in L* value.

**Table 1:** Means comparison of (A) formulation means over all temperatures and (B) temperature means over all formulations

**A**	0%CD	1%CD	3%CD	Pr>F
L*	89.97889a	89.96a	87.80444b	<0.01
Chrom	6.066667	5.954444	5.884444	0.22
Hue Angle	18.49333c	20.85222b	23.14444a	<0.01
Redness (a*/b*)	3.004a	2.635b	2.351c	<0.01
pH	3.31c	3.373333b	3.424444a	<0.01
Brix	16.27778b	16.13333c	16.87778a	<0.01
%water	4.455556b	4.711111b	5.088889a	<0.01
Water Activity	0.363444ab	0.341111b	0.376111a	<0.01
MPCTA	0.06435a	0.0089b	0.0568ab	<0.01

**B**	120°C	140°C	160°C	Pr>F
L*	88.86889	89.63111	89.24333	0.17
Chrom	6.183333a	5.816667b	5.905556b	0.01
Hue Angle	21.77222a	20.21667b	20.50111b	<0.01
Redness (a*/b*)	2.536b	2.716a	2.738a	<0.01
pH	3.372222	3.374444	3.361111	0.44
Brix	16.3b	16.35556b	16.63333a	<0.01
%water	5.788889a	4.611111b	3.855556c	<0.01
Water Activity	0.447222a	0.352889b	0.280556c	<0.01
MPCTA	0.092a	0.022b	0.017b	<0.01

a,b,c indicate significant differences between means based on Tukey's HSD adjustment to LS means.

**Table 2:** Means comparsion of all treatment variables

(a) Data before storage									
TRT	L‐value	Redness (a*/b*)	Chroma	Hue Angle	pH	Brix	Water Content	Water Activity	MPCTA (astringent)
120°–0%	89.9abc	2.79b	6.11ab	19.7bc	3.31a	16.2cd	6.07a	0.45b	0.13a
120°–1%	88.3abcd	2.61c	6.08ab	21.3b	3.37a	15.9d	5.03b	0.38c	0.03b
120°–3%	88.4bcd	2.21d	6.37a	24.3a	3.43a	16.8ab	6.27a	0.51a	0.12a
140°–0%	89.9abc	2.90b	6.02ab	19.1c	3.33a	16.2cd	4.10cd	0.38c	0.02b
140°–1%	91.0a	2.67c	5.76ab	20.4b	3.37a	16.0d	4.63bc	0.36c	0.00b
140°–3%	88.0cd	2.58c	5.67ab	21.2b	3.42a	16.9a	5.10b	0.32d	0.05b
160°–0%	90.2abc	3.33a	6.08ab	16.7d	3.29a	16.4bc	3.20e	0.26f	0.05b
160°–1%	90.5ab	2.62c	6.02ab	20.9b	3.38a	16.5bc	4.47c	0.29e	0.00b
160°–3%	87.0d	2.26d	5.62b	23.9a	3.42a	17.0a	3.90d	0.29de	0.00b
Pr>F	<0.01	<0.01	0.04	<0.01	0.42	0.03	<0.01	<0.01	<0.01

(b) Color data after storage				
TRT	L‐value	Redness (a*/b*)	Chroma	Hue angle
120°–0%	68.83ab	4.03bc	4.70a	14.02bcd
120°–1%	67.74ab	4.07bc	4.72a	14.05bcd
120°–3%	71.75a	2.53d	5.25a	21.62a
140°–0%	68.79ab	3.83bc	5.22a	14.68bcd
140°–1%	66.74ab	3.42bcd	5.03a	16.40bcd
140°–3%	65.97ab	4.30b	4.49a	13.10cd
160°–0%	64.27b	5.43a	4.55a	10.46d
160°–1%	69.16ab	3.42bcd	5.12a	16.41bc
160°–3%	69.42ab	3.24cd	4.75a	17.23b
Pr>F	0.03	<0.01	0.13	<0.01

TRT=drying temperature (°C) and % γ–cyclodextrin with the remainder as maltodextrin.

a, b, c, d, e indicate significant differences between means based on Tukey's HSD adjustment to LS means. *N* = 3.

The hue angle was generally less in the 0% γ‐cyclodextrin formulation than when 1% or 3% was added, indicating that the hue angle was more red than yellow with 0% γ‐cyclodextrin (Table 1). The hue angle of the 120°C formulation was more yellow than the 140°C and 160°C dried powders (Table 1), but the maltodextrin may have masked the red colored compounds slightly more at the lower drying temperature (Table 2a). Added γ‐cyclodextrin made the hue angle less red color and more yellowish (greater hue angle), or more susceptible to color masking. Hue angle color goes from reddish to yellowish as percent γ‐cyclodextrin increases and seems to be less affected by drying temperature (Table 2a).

Chroma was highest (color most vivid) in samples dried with 120°C inlet temperature (Table 1). Chroma was not significantly different between the three general means of the % γ‐cyclodextrin. At the higher drying temperatures of 160°C, the 3% γ‐cyclodextrin was least vivid (lower chroma) of all combinations (Table 2a).

Color of the spray‐dried powders changed after storage in all temperature‐formulation combinations. L‐value decreased (became darker), chroma decreased (became less vivid), hue angle decreased (moved toward red), and redness (a*/b*) increased. The 120°C with 3% γ‐cyclodextrin changed the least for three of the color values (L‐Value=16.63, hue angle=2.70, and redness (a*/b*) = −0.31). Meanwhile, the 160 °C with 0% γ‐cyclodextrin had the greatest change during storage for L‐Value (25.92), chroma (1.53), and redness (−2.10). In the 160 °C with 0% γ–cyclodextrin, hue angle did not result in the greatest decrease, but it was the lowest value at the beginning and after storage. (Table 2). It is unlikely that red‐colored compounds, such as antioxidants increased during storage, but it was more likely that browning occurred in the samples. If enough protein was available, Maillard reactions could have caused the darker color (Tonon, Brabet, & Hubinger, 2010). But, higher drying temperature did not cause darker powders in the beginning of the study, or after storage (Table 2), which would have been the case if Maillard browning occurred. Higher water activities (>0.43) can result in concentration of oxidized phenols and browning (Telis & Martínez‐Navarrete, 2010). The higher water activity of the 120°C dried samples tended to have the darker color after storage indicating that oxidation of phenols may have occurred.

### 3.2 Brix (Soluble solids)

Brix of reconstituted powders was greatest in the 3% γ‐cyclodextrin samples (Table 1). Brix increased at 160°C temperature over the 120°C and 140°C inlet temperatures. At each drying temperature, Brix was greatest at 3% γ‐cyclodextrin (Table 2) although it was the least at 1% γ‐cyclodextrin at lower drying temperatures of 120°C and 140°C. This may have something to do with the higher temperature‐spray‐dried powders not dissolving as much as the lower temperature‐dried powders.

### 3.3 Water content and water activity

Water content and water activity are greater in the powders dried at 120°C over the powders dried at 140°C and 160°C (Table 1). Obviously, the higher air temperatures resulted in more water removal. The formulations with the 3% γ‐cyclodextrin were greater in moisture content and water activity. This indicates that the γ‐cyclodextrin causes the powders to hold more water after drying, although the water activity of the 0% γ‐cyclodextrin was not significantly different from the 3% γ‐cyclodextrin (Table 1). Observing the individual treatment means in Table 2, the 0% γ‐cyclodextrin was least in water content when dried at 160°C, but 3% γ‐cyclodextrin was greatest when dried at 120°C, but not significantly different from 0% γ‐cyclodextrin mixture dried at 120°C (Table 2). Water activity generally followed a similar pattern as water content. However, the 160°C dried powder with 1% γ‐cyclodextrin was not different from the 3% γ‐cyclodextrin (Table 2) and the 0% γ‐cyclodextrin dried at 120°C was significantly different from the 3% cyclodextrin dried at 120°C.

### 3.4 pH

The pH of the reconstituted juice was greater or less acidic when the 3% γ‐cyclodextrin was included in the formulation over all the treatment temperatures (Table 1). Temperature did not significantly affect pH, and there was no interaction effect (Table 2).

### 3.5 MCPTA (astringency index)

MCPTA was greatest when no γ‐cyclodextrin was included, and was greater in the 120°C temperature spray‐dried samples (Table 1). The samples dried at 120°C with 0% γ‐cyclodextrin and 3% γ‐cyclodextrin were greater in MCPTA and more likely to be astringent or bitter than the other treatment combinations (Table 2) based on the relation of MCPTA and these flavor attributes observed by Bett‐Garber et al. (2014). But, based on the magnitude of the astringency index, the reconstituted spray‐dried powders probably had very low to no perceivable astringency or bitterness.

### 3.6 Correlation table

Both Hue angle and a*/b* are highly correlated with pH (Table 3). This indicates that pH directly impacted the red/yellow color of the powder and reconstituted solution. Although pH was not significantly affected by the drying temperature, it was affected by the γ‐cyclodextrin content. Hence, the higher pH made the powder less red. As was expected, hue angle negatively correlated with redness (a*/b*) (Table 3). Redness was lesser in the higher hue angle samples, whereas redness was greater in the lower hue angle samples. Hue angle and redness were both affected by inlet temperature and formulation and both measures are based on a‐value and b‐value on the color space. MCPTA was correlated with % water content and water activity (Table 3). It was interesting to note that as water content decreased, so did MCPTA astringency index. This may have something to do with inlet temperature effect that affects both measured in the same direction. Hue angle and L‐value (whiteness) were negatively correlated (Table 3). As the hue angle increased or became more yellow, the L* value (whiteness) tended to be less. This effect resulted because 0% γ‐cyclodextrin samples and the 1% γ‐cyclodextrin samples dried at 140°C and 160°C were more white than the 3% γ‐cyclodextrin samples, which were less white (lower L*values) and were more yellow (greater hue angle). Percent water content and water activity were predictably highly correlated (Table 3).

**Table 3:** Pearson correlation coefficients of dependent variables measured on spray dried juice

	L^a^ value	a^a^/b^a^	Chroma	Hue Angle	pH	Brix	Water Content	Water Activity
a^a^/b^a^	0.64							
Chroma	0.24	0.12						
Hue Angle	**‐0.68** a	**‐0.99** a	‐0.077					
pH	**‐0.66** a	**‐0.91** a	‐0.28	**0.91** a				
Brix	‐0.62	‐0.46	‐0.27	0.53	0.59			
Water Content	‐0.16	‐0.53	0.43	0.49	0.36	‐0.0012		
Water Activity	‐0.084	‐0.40	0.61	0.39	0.17	‐0.14	**0.87** a	
MPCTA	‐0.092	‐0.044	0.65	0.080	‐0.095	0.11	**0.72** a	**0.75** a

a Bolded Pearson correlation coefficients are significant.

### 3.7 Principle component analysis

**Figure 1:**
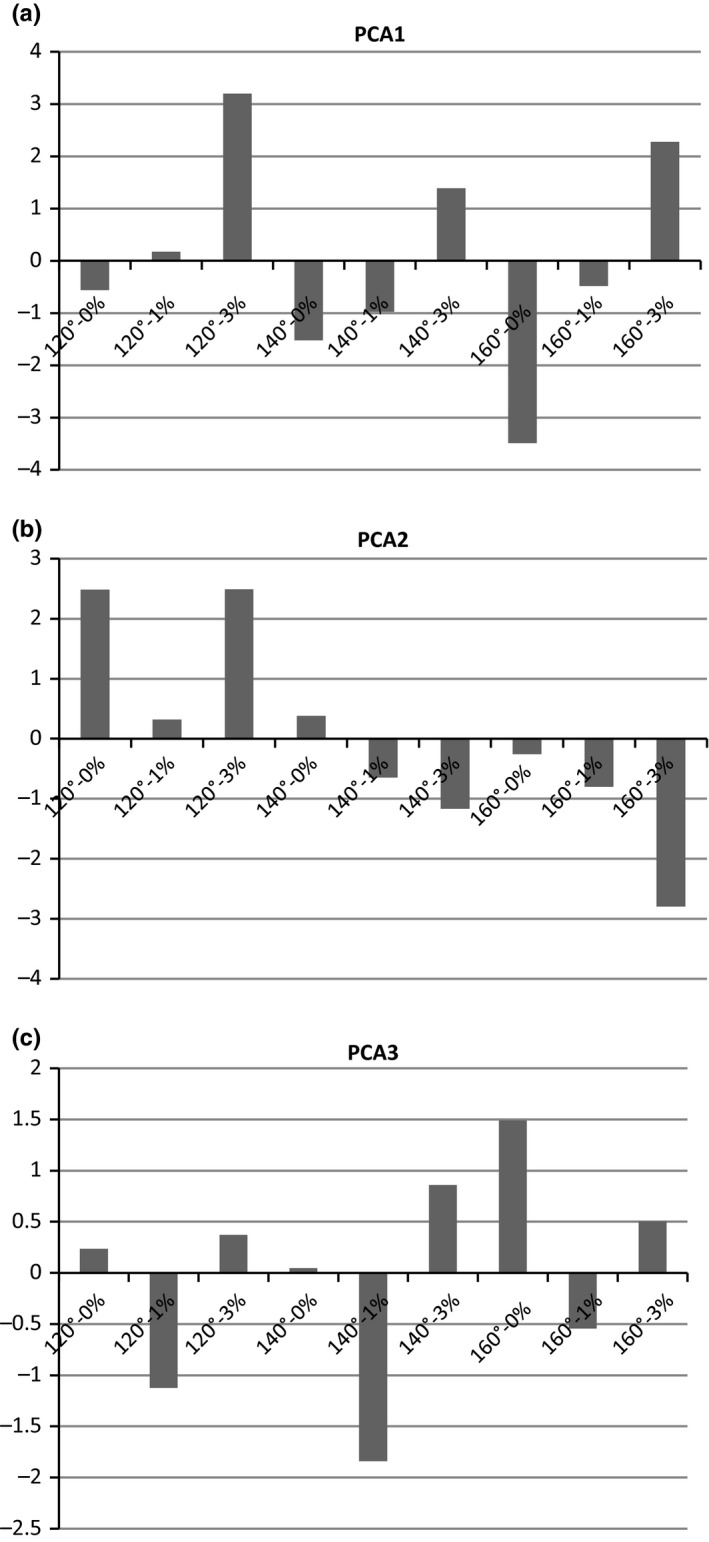
Principle component analysis results of how the dependent variables correspond to each other within the parameters of the juice formulations and drying methods. (a) pca 1–46.3% of variation, (b) pca 2–31.8% of variation, and (c) pca 3–11.6% of variation

The variables included in the principle component analysis (PCA) were L‐value, a*/b*, chroma, hue angle, pH, Brix, % water content, water activity, and MCPTA (astringency index). Three principle components accounted for 89.8% of the variation in the data. Component 1 was driven by L* value (eigenvalue of −0.37), a*/b* (eigenvalue of −0.47), hue angle (eigenvalue of 0.47), and pH (eigenvalue of 0.45); and accounted for 46.3% of variation. Component 1 was divided by formulation with the 3% γ‐cyclodextrin formulation on the positive side and the 0% γ‐cyclodextrin on the negative side (Fig. 1a). Hue angle and pH were positive with the 3% γ‐cyclodextrin, and L* value and redness (a/b) were negative with the 0% cyclodextrin. This supports what was discussed above, with hue angle and pH being greater in 3% γ‐cyclodextrin and less in the 0% γ‐cyclodextrin, while L* value and redness (a*/b*) were less in the 3% γ‐cyclodextrin and greater in the 0% γ‐cyclodextrin.

Component 2 accounted for 31.8% of the variation; and was driven by chroma (eigenvalue of 0.51), % water content (eigenvalue of 0.42), and water activity (eigenvalue of 0.50). Component 2 was divided by inlet air temperature (Fig. 1b). The 120°C temperature was on the positive side of the component, while the 160°C temperature treatments were on the negative side. Chroma, water content, and water activity were positive with the 120°C inlet temperature. Chroma, water content, and water activity were greater in the 120°C inlet temperature spray‐dried powders than in the 160°C spray‐dried powders. The 140°C inlet temperature was both positive and negative depending on the percent of γ‐cyclodextrin in the formulation.

Component 3 accounted for 11.6% of the variation and was driven by Brix (eigenvalue of 0.63) and MCPTA (eigenvalue of 0.56). Component 3 was divided by the 1% γ‐cyclodextrin on the negative side and the 0% and 3% γ‐cyclodextrin samples on the positive side (Fig. 1c). The 1% γ‐cyclodextrin tended to decrease the Brix and MCPTA slightly over the 0% and 3% γ‐cyclodextrin. Brix and MCPTA were in the positive space, while 1% γ‐cyclodextrin formulation was in the negative space. The 1% γ‐cyclodextrin samples were more susceptible to the inlet temperature, especially with regards to Brix and MCPTA.

## 4 Conclusions

The addition of γ‐cyclodextrin to the mixture of maltodextrin and juice results in a slightly yellow color instead of a reddish color. The addition of γ‐cyclodextrin to the mixture of maltodextrin and juice, also, increases the soluble solids (Brix). The addition of γ‐cyclodextrin tends to slightly raise the pH of the reconstituted solution, which relates to the change from red to yellow hue angle. The color of powders dried at 120°C were slightly more yellow than the powders dried at higher temperatures which were closer to the red hue angle. Principle component analysis of color, soluble solids, moisture content, water activity, pH, and astringency index resulted in 46.3% of the variation in the results being explained by the γ ‐cyclodextrin content and 32% was explained by the inlet air temperature. This means that the addition of y‐cyclodextrin with maltodextrin for spray drying had a slightly greater impact on color, moisture, pH, and astringency index than changes in spray drying inlet temperature.

## Conflict of Interest

None declared.
